# Portmanteau Constructions, Phrase Structure, and Linearization

**DOI:** 10.3389/fpsyg.2015.01851

**Published:** 2015-12-21

**Authors:** Brian Hok-Shing Chan

**Affiliations:** Department of English, Faculty of Arts and Humanities, University of MacauMacau, China

**Keywords:** code-switching, portmanteau construction, word order, phrase structure, linearization

## Abstract

In bilingual code-switching which involves language-pairs with contrasting head-complement orders (i.e., head-initial vs. head-final), a head may be lexicalized from both languages with its complement sandwiched in the middle. These so-called “portmanteau” sentences (Nishimura, 1985, 1986; Sankoff et al., 1990, etc.) have been attested for decades, but they had never received a systematic, formal analysis in terms of current syntactic theory before a few recent attempts (Hicks, 2010, 2012). Notwithstanding this lack of attention, these structures are in fact highly relevant to theories of linearization and phrase structure. More specifically, they challenge binary-branching (Kayne, 1994, 2004, 2005) as well as the Antisymmetry hypothesis (ibid.). Not explained by current grammatical models of code-switching, including the Equivalence Constraint (Poplack, 1980), the Matrix Language Frame Model (Myers-Scotton, 1993, 2002, etc.), and the Bilingual Speech Model (Muysken, 2000, 2013), the portmanteau construction indeed looks uncommon or abnormal, defying any systematic account. However, the recurrence of these structures in various datasets and constraints on them do call for an explanation. This paper suggests an account which lies with syntax and also with the psycholinguistics of bilingualism. Assuming that linearization is a process at the Sensori-Motor (SM) interface (Chomsky, 2005, 2013), this paper sees that word order is not fixed in a syntactic tree but it is set in the production process, and much information of word order rests in the processor, for instance, outputting a head before its complement (i.e., head-initial word order) or the reverse (i.e., head-final word order). As for the portmanteau construction, it is the output of bilingual speakers co-activating two sets of head-complement orders which summon the phonetic forms of the same word in both languages. Under this proposal, the underlying structure of a portmanteau construction is as simple as an XP in which a head X merges with its complement YP and projects an XP (i.e., X YP → [_XP_ X YP]).

## Introduction: The portmanteau construction in bilingual code-switching

This paper seeks a new account of a specific construction in bilingual code-switching which has so far received few in-depth treatments and remains not well-understood, based on existing data gleaned from all works that are accessible, including published papers and unpublished dissertations. The portmanteau construction in code-switching, which involves the juxtaposition of two synonymous morphemes from two different languages^1^, has been attested in various datasets for decades (Nishimura, 1985, 1986; Park, 1990; Sankoff et al., 1990), but nonetheless there had been no systematic studies of the construction (that I know of) until quite recently (Hicks, 2010, 2012). The form of the portmanteau construction is sketched below.

(1)   [_XP_ X_A_ [YP (ZP)] X_B_]

     (Language A is head-initial in XP whereas language B is head-final in XP)

(Language A is head-initial in XP whereas language B is head-final in XP)

X, the doubled element, is a head, whereas the shared element is the complement of this head, namely, YP. In some cases, a head, such as a ditransitive verb, may select two complements, hence YP and ZP^2^. The languages which participate in the portmanteau construction (i.e., A and B) are mostly typologically different with one (say, language A) being a VO language and another (say, language B) being an OV language, for instance, Japanese-English (Nishimura, 1985, 1986; Azuma, 1993, 1997, 2001; Takagi, 2007; Furukawa, 2008; Namba, 2012a,b), Korean-English (Park, 1990), Hindi-English (Pandit, 1986), Tamil-English (Sankoff et al., 1990), or Marathi-English (Hicks, 2010, 2012), etc. Some data of the portmanteau construction are also attested in a pair of an OV language and a partially VO language (e.g., Dutch-Turkish in which Dutch is VO in main clause but OV in subordinate clause—see Backus, 1996, 2003), or two SVO languages (e.g., Cantonese-English in which Cantonese is postpositional but English is prepositional—see Chan, 1998, 2015).

It appears that in such constructions the doubled element is often the verb. For Nishimura (1995, p. 167), “‘[p]ortmanteau sentences’ involve a specific type of repetition: an English sentence and its Japanese equivalent are combined with a commonly-shared constituent. Portmanteau sentences come out in SVOV order: O is the common constituent. The first V is English, and the final V is Japanese.” The following are some examples in which the doubled element is a verb whereas the shared element is an object DP.

(2)   We **bought** [about two pounds *gurai*] ***kaettekita*** *no*

                                                        about bought     PRT

        “We bought about two pounds.”

                              (English-*Japanese*, Nishimura, 1986, p. 139)

(3)   One day my friend **brought** [two watch] *kaciyo*

                                                                      have

                 ***wasseyo***

                 come (= bring)

        “One day my friend brought two watches.”

                                         (English-*Korean*, Park, 1990, p. 103)

There are some data which look like examples (2)–(3), such as (4) and (5) below, but the two verbs actually carry quite different meanings. Strictly speaking, they do not involve the same word being lexicalized in two languages, and therefore they are considered very different and excluded from the present account.

(4)   I still have [*etten namca*] *coha-hayyo*

                        certain man   like

        “I still have a certain man that I like.”

                                          (English-*Korean*, Park, 1990, p. 103)

(5)   You pull [this much] *tsukau desho*

                                      use       will

        “You pull this much that you'll use.”

                                (English-*Japanese*, Nishimura, 1986, p. 139)

The shared element of a doubled verb may be more complex than just an object DP. In (6) there are two objects since the doubled verb is ditransitive “*give.*” In (7) the shared element is a PP.

(6)   They **gave** [me] [a research grant] ***koDutaa***

                                                           give (3-Sg-Past)

        “They gave me a research grant.”

                                     (English-*Tamil*, Sankoff et al., 1990, p. 93)

(7)   I was **talking** [to *oru orutanooDa*] ***peesinDu***   *irunten*

                                   one person            talk-PROG be(1SG-PAST)

        “I was talking to one person.”

                                    (English-*Tamil*, Sankoff et al., 1990, p. 93)

Some verbs take clauses as complements, and it is not surprising to find the following examples in which the shared element is a CP.

(8)   Everybody **think** [**that** *nay-ka yenge-lul     cal   hanta-**ko***]

                                    C     I-NOM English-ACC well do-C

                  ***sayngkakhayyo***

                  think

            “Everybody thinks that I'm a good English speaker.”

                                             (English-*Korean*, Park, 1990 p. 103)

(9)   Many people **told** [me] [**that** *cey-ka  hankwukcek-ita*-***ko*]**

                                                     I-NOM Korean-oriented-C

                  ***malhaysseyo***

                  told

            “Many people told me that I am Korean-oriented.”

                                             (English-*Korean*, Park, 1990, p. 103)

Whereas, the complementizer is also doubled in examples (8) and (9) above, sometimes the complementizer is phonetically realized in one language only, as in (10) below.

(10)  I **think** [it's the European influence-*nu*] ***ninakiren***

                                                             that think (I-SG-PRES)

         “I think that it's the European influence.”

                                   (English-*Tamil*, Sankoff et al., 1990, p. 92)

The examples given so far contain lexical verbs. Apart from these verbs, the copula verb “*be*” also takes part in portmanteau sentences, as in examples (11)–(14) below.

(11)  Dus in Nederland **zijn**     [zoveel devlet hastanesi]

        So   in Holland     are-3PL so-many state hospital

                   ***var***

                   there-are

                “so in Holland there are so many state hospitals…”

                                        (Dutch-*Turkish*, Backus, 1996, p. 348)

(12)  It **was** [*cengmal* exiting game] **-*iyesseyo***

                           really                             COP-PAST

         “It was really an exciting game.”

                                           (English-Korean, Park, 1990, p. 103)

(13)  There'**s** [children] ***iru*** *yo*

                                    V   (existential)

        “There are children.”

                              (English-*Japanese*, Nishimura, 1985, p. 140)

(14)  She will not come to me because the hindu system **is**

         [*tarah kaa]* ***hai***

         that     of      is

         “She will not come to me because the Hindu system is like that.”

                                            (English-*Hindi*, Pandit, 1986, p. 41)

An auxiliary verb can also be doubled, such as (7) above (“*was*” in English and “*irunten*” from Tamil). A similar example is (15) below, where the doubled element is an auxiliary verb cliticized with the negation marker, and the shared element is a verb or a verb phrase.

(15)  My parents **didn't** [*helak-haci*] ***anasseyo***

                                     allow-do       V+NEG

         “My parents didn't allow (me to do it).”

                                            (English-*Korean*, Park, 1990, p. 104)

The doubled element is not necessarily a verb or an auxiliary verb. Examples (8) and (9) above have already shown that a complementizer may be doubled. In (16) below, it is a subordinator, which is similar to a complementizer in taking a TP or IP complement, that is being doubled.

(16)  Just **because** [*avaa innoru*   color and race]

         just because they   different color and race

                   ***engindratunaale***

                   of-because

                “Just because they are of different color and race.”

                                     (English-*Tamil*, Sankoff et al., 1990, p. 93)

In examples (17–20), the doubled element is an adposition.

(17)  I could run every you know **in** [thirty minutes]

                  ***madhe*** once a day.

                  in

         “I could run every, you know, in thirty minutes once a day.”

                                          (English-*Marathi*, Hicks, 2010, p. 45)

(18)  Look for the things she buys **for** [Sean] ***ni***

                                                                     for

         “Look for the things she buys for Sean.”

                                 (English-*Japanese*, Nishimura, 1986, p. 140)

 (19) **According to** [the schedule] ***paDi***

                                                    according-to

         “According to the schedule… ”

                                     (English-*Tamil*, Sankoff et al., 1990, p. 93)

(20)  **After** [*ni1   -go3* review] ***zi1-hau6***…

                  DEM CL             after

         “After this review…”

                                       (English-*Cantonese*, Chan, 1998, p. 204)

Referring back to the Japanese-English example (2), there is an English preposition “*about*” that is coupled with the Japanese one “*gurai*” in the object “*about two pounds gurai.*” The prepositions here, however, do not act as prototypical prepositions that mark the location or semantic role of a DP, but rather they somehow modify a noun phrase that denotes an object with a quantity. Accordingly, they look like a “pre-determiner” in traditional descriptive grammar. In generative grammar, they are most probably not instantiations of a P category but more likely of a functional head in the D domain, probably a quantifier head Q. The following is another example, also from Japanese-English, in which a “pre-determiner,” probably a Focus head F, is doubled.

(21)  Vegas *it-tara dare*     **even** [the tour leader]

                  go-if   anyone

                  ***demo*** they don't lend him money

                  even

         “If you go to the Vegas, even the tour leader doesn't lend him money (if somebody has been robbed).

                              (English-*Japanese*, Furukawa, 2008, p. 286)

Summarizing this survey of portmanteau constructions, we see that heads which take part in the construction include verb, auxiliary verb, preposition (or adposition), complementizer, subordinator, and some functional heads in the DP domain. Other categories which act as syntactic heads, including noun, adjective, modal verb or conjunction [with a possible exception in (23), see below] have not been found in existing data of portmanteau constructions (see more discussion below).

In all corpora in which the portmanteau construction is found, the bilingual speakers also produce non-portmanteau code-switched constructions; that is, these heads do not have to be doubled [see Chan, 2003, 2008 for a quick survey, also see (39)–(44) below]. In other words, the portmanteau construction is an optional structure.

Repetition involves not only words or free morphemes but also bound morphemes.

(22)  …dzimwe dzenguva tinenge tichiita **ma***-game-****s*** panze

         “**…** sometimes we will be doing games outside.”

                              (Shona-*English*, Myers-Scotton, 1993, p. 132)

Repetition may also take place with a word (or free morpheme) and a bound morpheme which are synonymous. In (23) below, the Spanish conjunction “*pero*” is doubled with “*sti*” from Aymara, which appears to be a bound morpheme affixed to nouns^3^ (Hicks, 2010, p. 16).

(23)  **pero** *sorro*- ***sti***                  *wali astuturi- tajna*…

         but   fox      COORDINATOR very keen       3SG.PRT.EVI

         “But the fox was very keen…”

                                          (Spanish-*Aymara*, Stolz, 1996, p. 10,

                                                          cited in Hicks, 2010, p. 16)

Covering phenomena illustrated by (22) and (23), Hicks (2010, 2012) counts the portmanteau construction as one type of “morphosyntactic doubling.” This paper acknowledges that the process underlying the doubling in (22) and (23) may be very similar to that underlying the doubling of words as shown in examples (2)–(3) and (6)–(21) above. In particular, as a syntactic head combines with its complement in a fixed order (i.e., head-initial or head-final), a bound morpheme is always attached to a root of a particular category (e.g., a plural affix is always attached to a noun, etc.) in a fixed order (i.e., prefix, suffix, or the more uncommon infixes). There are also differences awaiting explanation^4^, and existing data of “morphological doubling” (i.e., two synonymous bound morphemes from two languages) are extremely rare, namely, a few instances from Myers-Scotton (1993) as quoted in Hicks (2010, 2012) and marginally (23) above. Hence, this paper focuses on the portmanteau construction or syntactic doubling in code-switching (i.e., two synonymous words or free morphemes from two languages), which does not deny the possibility of pursuing a uniform account of “morphological doubling” and “syntactic doubling” when more data of the former are uncovered.

The remaining parts of this paper are structured as follows. The next section discusses differences between portmanteau constructions and monolingual syntactic doubling. The following one proposes constraints on the portmanteau constructions. These constructions are then tested against current paradigms of the syntax of code-switching and more general syntactic theories of phrase structure (e.g., Antisymmetry). A new account based on syntax and processing will then be forwarded, followed by a discussion of some residual issues and the conclusions.

## Portmanteau constructions and monolingual doubling phenomena

Putting aside morphology, the term “syntactic doubling” may not be entirely appropriate in describing the portmanteau constructions in code-switching, since “syntactic doubling” may refer to some monolingual phenomena (Barbiers, 2008; Barbiers et al., 2008) which, as Hicks (2010) cogently argues, are very different in nature. The following are sampled from the phenomena discussed in the volume of Barbiers et al. (2008).

(24)  **An** a   **han** joort hi

         He has he  done it

        “He has done it.

                                     (Finland Swedish, Barbiers, 2008, p. 11)

(25)  He **should can** go tomorrow.

                                       (Scottish English, Barbiers, 2008, p. 16)

(26)  Jan **kan** best schaatsen **kunnen**

        Jan can  best skate        can.INF

        “It is perfectly possible that John is able to skate.”

                                                    (Dutch, Barbiers, 2008, p. 17)

(27)  Ek ken    **nie** daardie man **nie**

         I   know not that        man not

        “I don't know that man.”

                                           (Afrikaans, Biberauer, 2008, p. 104)

(28)  **ä** ganz  **ä**  liebi  frau

         a really a lovely wife

         “a really lovely wife”

                                           (Swiss German, Barbiers, 2008, p. 5)

(29)  **Leer**,       ningún estudiante ha  **leido** este libro

         Read-INF no       student    has read this book

         “As for reading, no student has read this book.”

                                                  (Spanish, Barbiers, 2008, p. 16)

(30)  **Um**   hvað eruð þíð     að tala **um**

         about what are you-PL to talk about

         “What are you talking about?”

                                               (Icelandic, Jónsson, 2008, p. 404)

Doubling of pronouns such as (24) is distinct from the portmanteau construction in the sense that the doubled pronouns have no complements. In (25), however, the two modal auxiliary verbs (i.e., “*should*” and “*can*”) share a complement VP (“i.e., “*go tomorrow*”). In a sense the syntactic category of modal verb, presumably a functional head in the I or T domain, is doubled, but the modals here are two different words of two different meanings. In portmanteau sentences, the doubled heads appear to be of the same word though realized in two different phonetic forms associated with two separate languages. In (26), the doubled modal auxiliary is of the same word [“*kan* (can)”], and in terms of surface order this example looks very similar to a portmanteau construction in which two instances of “*kan* (can),” supposedly an I or T head again, surround a complement VP [“*best schattsen* (best skate)”]. However, as shown in the English translation and explained by Barbiers (2008, p. 17), the two instances of “*kan*” convey quite different meanings; that is, the first one is epistemic and has scope over a proposition (i.e., *It is* ***possible*** *that PROPOSITION)* whereas the second one denotes the subject's ability (i.e., *John **is able to** skate*). In portmanteau sentences, the doubled heads appear to carry essentially the same meaning^5^. In (27), the doubled negation markers do seem to convey the same meaning, but, as Biberauer (2008) explains, only the first “*nie1*” is a NEG head merged in VP, whereas the second one, “*nie2*,” is really a Polarity Head above CP (that dominates the VP). The first “*nie1*” moves up to the specifier position of the Polarity phrase with VP, resulting in the “*nie1* VP *nie2*” sequence. This movement account does not extend to portmanteau sentences, if we assume that the doubled head (X_A_ and X_B_) is of the *same* syntactic category (e.g., V, C, T, or P)^6^.

In (28), the doubled indefinite determiners do seem to be the same word conveying the same meaning. Nonetheless, the first one, which is optional, is licensed by a degree or quantity expression [e.g., “*ganz* (really),” also see Kallulli and Rothmayr, 2008]. In portmanteau sentences, neither instance of the doubled heads seems to be licensed by an element other than its complement (i.e., the complement is obligatory in a portmanteau sentence). In (29), again, the doubled heads, which is a verb in this case, are the same word with the same meaning, but the first one [“*leer* (read)”] carries an intransitive reading whereas the second one [“*leido* (read)”] is transitive (Barbiers, 2008). In portmanteau constructions, both verbs are transitive and argument-sharing. In (30), the doubled prepositions “*um* (to)” do share the same complement [i.e., “*hvað* (what)”], but the second one is far away from the complement which has undergone wh-movement (Jónsson, 2008). In portmanteau sentences, both instances of the doubled head are both contiguous to their complement.

Having pointed out the differences between the portmanteau construction as a kind of “syntactic doubling” and “syntactic doubling” in monolingual phenomena, it would be fair to mention that in fact the names “portmanteau” (Nishimura, 1995, p. 157) and “palindromic switches” (Sankoff et al., 1990, p. 52) are not necessarily better descriptions of the code-switched construction being discussed. The term “portmanteau” is supposed to refer to “blends” originally (e.g., “*smog*” that is blended from “*smoke*” and “*fog*”)^7^. Portmanteau constructions in code-switching obviously do not refer to such lexical blends but they are more like “syntactic blends” (e.g., SVOV is blended from SVO and SOV). “Palindrome” denotes a series of linguistic items, including alphabets or words, which is the same whether reading forward or backward, such as “*madam*”^8^. Again, the portmanteau sentences are palindromic only in the sense of their syntactic sequence (i.e., X YP X). This paper adheres to the name of “portmanteau” because it is deemed a more popular one for the code-switched construction being examined.

This comparison with monolingual syntactic doubling and discussion of the names (i.e., “doubling” vs. “portmanteau” vs. “palindromic”) is cursory and by no means comprehensive^9^, but hopefully it serves to sharpen our focus on the so-called portmanteau construction in code-switching. In particular, we are dealing with cases of “lexical doubling” where the same word is realized into two synonymous but different phonetic forms. Of course, there is also “syntactic doubling” at the same time; that is, two words of the same category (i.e., X_A_ and X_B_) appear in the sentence. However, the fact that these heads appear in positions adjacent to and on both sides of their shared complement seems better captured by the descriptors of “portmanteau” or “palindromic.”

## Constraints on the portmanteau construction

As commented by Sankoff et al. (1990, p. 52), “[p]alindromic switches, also known as portmanteau, copy translations, or mirror-image constructions, are widely attested but are inevitably found to occur rarely in quantitative studies.” They continued, “Thus, these seem to constitute an occasional *ad hoc* production strategy rather than a systematic approach to bilingual sentence production” (Sankoff et al., 1990, p. 52).

Whereas, the portmanteau construction is indeed rare or unexpected in relation to not only monolingual phenomena but also code-switching, these data should not be automatically brushed aside as “periphery” (vs. “core,” Chomsky, 1981) or performance data for which any attempt of systematic explanation is deemed futile. Crucially, portmanteau sentences have been attested in disparate speech communities and in different datasets, involving various language-pairs. It is at least a recurrent pattern in code-switching which is predictable in code-switching with typologically different languages. Additionally, in these language-pairs, portmanteau constructions are a general pattern which involves not only lexical verbs but also different kinds of heads (see above for a brief survey and see below for more details). Last but not least, it is clear that there are syntactic patterns or regularities that are amenable to more general explanation in terms of syntactic constraints, particularly the following.

(31)  *Some heads do not double*.

The first regularity concerns the lack of data in which nouns, adjectives, modals, and conjunctions act as the doubled head in portmanteau constructions. It is not entirely clear whether the absence of these categories is due to empirical gaps (i.e., they are possible but they have not been attested) or some syntactic reasons. Worse still, grammaticality judgment, which potentially differentiates both scenarios, is not always reliable or consistent for code-switching since it may be affected by varying bilingual proficiency (MacSwan, 1999; Toribio, 2001), not to mention the irregularity of the portmanteau constructions under examination^10^. Based on the available data, tentatively speaking, the absence of modals or determiners may be just an empirical gap, if auxiliary verbs (e.g., (15), also supposed to be in I/T as modals) or some other functional heads in DP [e.g., (2), (21)] can be doubled in portmanteau constructions. On the other hand, there may be more deep-rooted reason underlying the absence of nouns (predicative) adjectives^11^ and conjunctions.

Nouns are not found to partake in portmanteau constructions in language-pairs where the “noun complements” canonically appear on different sides of the head noun, such as Cantonese-English (Chan, 2008, 2015), Hindi-English (Pandit, 1986), or Tamil-English (Sankoff et al., 1990). In earlier frameworks such as X-Bar Theory (Jackendoff, 1977), nouns do take complements; for instance, a derived nominal or nominalization takes a DP complement [e.g., (32a)], see Chomsky, 1970), similar to the way in which its related verb takes an object [e.g., (32b)]. However, contrary to objects of transitive verbs which are obligatory, noun complements are grammatically optional [e.g., (32c)].

(32)  a. the destruction of Rome

                  N               P DP

                  HEAD           COMPLEMENT

         b. The barbarians destroyed Rome.

                                     V             DP

                                     HEAD     COMPLEMENT

         c. The destruction caused a lot of casualties.

                    N (HEAD)

Another difference between nouns and verbs is that nouns cannot take their complement directly. In Government-and-Binding Theory, this is because nouns lack case-assigning properties (Chomsky, 1981). To introduce its complement, a case-assigner has to be introduced, such as a preposition in English or a “nominalizer” in Chinese languages which is most likely a functional head. Such nominalizers or genitive markers are attested in other languages where the “noun-complements” are prenominal, such as “*ke*” in Hindi (Pandit, 1986) or “*uDaya*” in Tamil (Sankoff et al., 1990).

(33)  lo4-maa5 **ge3**   mit6-mong4

        Rome       NOM destruction

        “The destruction of Rome”

                                                (Cantonese)

This case-based account, however, does not explain “*that*” which is required to introduce sentential complements of nouns (e.g., (34)—Haegeman and Guéron, 1999, p. 440); these sentential complements are not supposed to bear case.

(34)  The news ^*^(that) Peter has resigned bothered me.

               N                 CP

               HEAD          COMPLEMENT

In view of the optionality of the so-called “noun-complements,” Kayne (2009) proposes that they are in fact a variety of relative clauses, which are an adjunct rather than a complement. In other words, nouns actually do not take complements. If this is on the right track, then it is not surprising at all that nouns do not take part in portmanteau constructions, an integral condition for which is that a head merges with its complement and projects a phrase with the same label.

Not much is known about the case of adjectives. Attributive adjectives are standardly analyzed as an adjunct or a specifier of a functional head in the Cartographic Approach to syntax (e.g., Cinque, 2005) which does not take a complement. Some predicative adjectives do seem to license internal arguments but at least in English they do not take them directly; similar to the case of nouns, a preposition is called for to introduce a complement.

(35)  The manager is open^*^ (to) different suggestions.

                                ADJ    P  DP

                                HEAD     COMPLEMENT

Pending confirmation from further research, it is plausible that at least in some languages (e.g., English) adjectives do not project to an Adjective Phrase with a complement either. If this were a more general phenomenon across languages, an adjective would not take part in portmanteau constructions, even though it might canonically appear on both sides of its internal argument [e.g., “*different suggestions*” in (35)] in the languages that a bilingual speaks. At any rate, there seems little existing data of code-switching which involve two languages in which predicative adjectives show different head-complement order.

Assuming that conjunctions are a functional head on a par with complementizers (C), determiners (D) and *do*-auxiliary verbs, which take part in the portmanteau construction, we expect to find conjunctions being doubled in a portmanteau construction too. Contrary to expectation, there are few instances of the portmanteau construction involving a doubled conjunction [except (23) above in which one conjunction is an affix attached to the subject noun]. One possible reason is that a conjunction rarely appears after the second conjunct clause (i.e., [XP CONJ YP] is possible but [XP YP CONJ] is much rarer)^12^. At any rate (as far as I am aware of) there is not any attested evidence of code-switching between a language that licenses an [XP CONJ YP] order and another that allows an [XP YP CONJ] order.

The absence of a sequence of [XP YP CONJ] is very much a logical consequence if we subscribe to Chomsky's (2013, p. 46) recent suggestion that CONJ does not merge with a conjunct clause (i.e., XP or YP) but a sequence of [XP YP]. Failure to label the phrase [XP YP] drives the movement of XP above CONJ, resulting in [XP CONJ XP YP] which is labeled as an XP but not a CONJP. In such an account, CONJ is not a projecting head in the sense that it does not first-merge with its complement (e.g., YP) and project a phrase (i.e., ^*^CONJ YP → [_CONJP_ CONJ YP]).

(36)  *Complements do not double*.

A second recurrent pattern is that it is the head that is doubled, but never (to the best of my knowledge) are there data in which the complement is doubled rather than the head. If this possibility sounds outlandish, we may be reminded that in the minimalist program all derivations are possible unless they are “crashed” for some reason (Chomsky, 1995; MacSwan, 1999). In other words, the impossibility of a [YP X_A∕B_ YP] sequence calls for an explanation.

The absence of an SOVO pattern may be explained by the classic theta-criterion in the earlier Government-and-Binding Theory^13^.

(37)  *The Theta-Criterion* (Chomsky, 1981, p. 36)

         a. Each theta role is assigned to one and only one argument.

         b. Each argument is assigned one and only one theta role.

In accordance with (37a), one object in an SOVO structure would not receive a theta/thematic role, hence the impossibility of such a sequence. On the other hand, although the subject and object in an SVOV structure apparently receive theta-role twice from the two reduplicated verbs, these reduplicated verbs are arguably the *same* word (see above) and hence also the *same* verb, and supposedly the subject and the object still receive one and *the same* role (e.g., Agent for Subject, Theme for Object, Recipient for the Indirect Object, etc.)^14^. However, this explanation cannot account for other types of portmanteau constructions in which the doubled element are from other categories which do not assign theta-roles (e.g., copula verb or auxiliary verb).

(38) *Word order (i.e., head-initial vs. head-final) always follows the language of the head*

A third regularity is that the head from head-initial language (e.g., X_A_) always remains head-initial whereas that from the head-final language (e.g., X_B_) always stays head-final in the portmanteau sentences. Whereas, this sounds self-evident or merely descriptive, this regularity is not to be taken for granted particularly in SVOV structures, since in non-portmanteau, code-switched sentences a verb from a head-initial language can appear in head-final position, and in reverse a verb from a head-final language can also appear in head-initial position. The following are some examples from various typologically different language-pairs surveyed in Chan (2003, 2008, 2009).

V from OV language, VO order

(39)  **pooTuruvaan                   *letter***

         put (3-SG-MASC-FUT) letter

         “He will write a letter.”

                                    (Tamil-*English*, Sankoff et al., 1990, p. 79)

(40)  I have to ***ttak*ē my hand**

         I have to wash my hand

         “I have to wash my hand.”

                                           (English-*Korean*, Choi, 1991, p. 889)

V from OV language, VO order

(41)  i      ka      **i**       ***rectify***

        3PL TAM 2SG rectify

         “They usually rectify you.”

                   (Mandinka-*English*, Haust and Dittmar, 1997, p. 88)

(42)  want      on   Tex laat ons **daai (daardie) *group join***

         because old  Tex make 1PL DEM          group join

         “Because old Tex made us join that group.”

               (Tsotsitaal-*English*, Slabbert and Myers-Scotton, 1996,

                                                                                        p. 332)

The following two examples also show OV order with a verb from a VO language, but they involve the so-called “mixed compound verb” structure in which an auxiliary verb “*do*” appears in I/T (Chan, 2003, 2008)^15^.

(43)  kamalaa ne    hamaare ghar   par ***chicken taste*** kiyaa

         Kamla   ERG our        house at  chicken taste did

         “Kamla tasted chicken at our house.”

                                            (Hindi-*English*, Pandit, 1986, p. 106)

(44)  anta ***car*-ei      *drive*** paNNanum

         that car-ACC drive do + must

         “We must drive that car.”

                                   (Tamil-*English*, Sankoff et al., 1990, p. 80)

Such “mismatch” between the language of the verb (i.e., VO or OV) and the order of the code-switched phrase, however, does not extend to other heads. From available data, the language of a functional head, including adpositions, always determines head-complement order in code-switching, either in portmanteau and non-portmanteau sentences (e.g., English preposition always remains prepositional and a Cantonese postposition always remains postpositional in a code-switched PP—Chan, 2015).

## Portmanteau constructions and syntactic models of code-switching

Neither are the form of portmanteau constructions and the constraints on them captured by current syntactic models of code-switching (Hicks, 2010, 2012), including the Equivalence Constraint (Poplack, 1980), the Matrix Language Frame Model (Myers-Scotton, 1993, 2002; Myers-Scotton and Jake, 2009), the Bilingual Speech Model (Muysken, 2000, 2013) and the Null Theory (Mahootian, 1993; MacSwan, 1999, 2000; Chan, 2003, 2008).

As a classic that stimulated much subsequent work on the syntax of code-switching, the Equivalence Constraint (Poplack, 1980) prohibits code-switching at points where the surrounding words have divergent word orders in the participating languages (Poplack, 1980, p. 228; Sankoff and Poplack, 1981, p. 5–6). Accordingly, with switches between head and complement within a phrase (e.g., DP, VP, PP, or CP) whose word orders contrast in the participating languages (i.e., head-initial vs. head-final), portmanteau constructions violate the Equivalence Constraint (Poplack, 1980; Hicks, 2010, 2012)^16^. Looking at Tamil-English, which is one major source of the portmanteau data, Sankoff et al. (1990, p. 92) acknowledge the violation. However, they think that these constructions are a way to circumvent the Equivalence Constraint since the word orders of both languages are respected “as the lesser of evils” (Sankoff et al., 1990, p. 92). This idea is sensible, but counter-examples of the Equivalence Constraint in data other than portmanteau constructions (see Chan, 2003 for a survey) weaken the validity of the constraint and the feasibility of this suggestion.

If the Equivalence Constraint is unrealistically too narrow in confining code-switching to where two grammars overlap in bilingual competence (Poplack, 1980, p. 612, Figure 3; Sankoff and Poplack, 1981; Woolford, 1983), the Matrix Language Frame Model (Myers-Scotton, 1993, 2002) is certainly broader in empirical scope, but nonetheless there is a baseline. That is, the grammar of the more dominant language, that is, the Matrix Language (or ML), has to be observed. This is supposed to be the case since the Matrix Language alone constructs the “frame” of a code-switched sentence (via *the Uniform Structure Principle*—Myers-Scotton, 2002, p. 8–9). There are two ways in which this is accomplished. One, ML sets the word order of a code-switched sentence via *the Morpheme Order Principle*, and, two, ML provides the “system morphemes,” mostly function words or bound morphemes, via *the System Morpheme Principle*. The less dominant language, namely, the Embedded Language, only contributes content words (or “content morphemes”) or phrases (i.e., “EL islands” which nonetheless are formed in EL grammar by virtue of the *EL Island Principle*) to be inserted into the frame. Being another paradigm in the syntax of code-switching, there has been much follow-up discussion and extension of the original model (Myers-Scotton, 2002, 2006; Myers-Scotton and Jake, 2009), in particular, the appended “4M” Model. It proposes a more fine-grained classification of system morphemes so that the “early system morphemes” (such as determiners or plural suffixes) may be activated from EL but the “bridge” morphemes (e.g., the non-theta assigning preposition “*of* ”) or the “outsider” morphemes (e.g., agreement markers) are rarely accessed from EL (Myers-Scotton, 2002, 2006; Myers-Scotton and Jake, 2009).

Concerning the portmanteau construction, it is sufficient to note that the juxtaposition of word orders from both languages in a code-switched sentence makes it impossible to designate the Matrix Language and hence also the Embedded Language in that sentence, since the design of the model requires that one participating language has to be the ML and the other be EL (in accordance with *the Asymmetry Principle*, see Myers-Scotton, 2002, p. 9). In other words, the Morpheme Order Principle has to be violated by the portmanteau constructions; to be more concrete, referring back to (1), if language A were the ML, the order of YP X_B_ would be against the word order of the ML; if language B were the ML, the order of X_A_ YP would contradict the word order of ML. Also challenged are the Asymmetry Principle (which dictates that one language is ML and the other is EL) and the Uniform Structure Principle (which stipulates that ML alone contributes to the structure of a code-switched constituent—Myers-Scotton, 2002, p. 8–9)^17^.

In a theoretical perspective, the Matrix Language Frame Model may be too “heavy” in invoking a “grammar” specific to code-switching (Chan, 2003, 2008, 2009). In this light, greater theoretical and cognitive economy is achieved in attempts that subsume recurrent patterns in code-switching into general constraints independently proposed for monolingual phenomena, an early one being the Government Constraint (DiSciullo et al., 1986). The idea that code-switching and monolingual sentences are governed by the same linguistic constraints and mechanisms has eventually been dubbed “the Null Theory” since Mahootian (1993), inspiring later works (MacSwan, 1999, 2000; Chan, 2003, 2008, also see MacSwan, 2014). Strictly speaking, the Null Theory is *not* one coherent theory but more of a theoretical position, and studies that claim to follow “the Null Theory” may make different empirical predictions because of the various syntactic theories or constraints they appeal to respectively [e.g., Tree-Adjoining Grammar for Mahootian (1993), The Principles-and-Parameters Framework for Chan (2003, 2008), or the Minimalist Program for MacSwan (1999, 2000) and the papers in MacSwan (2014), see Chan, 2009 for a summary]. Not surprisingly, the term “Null Theory” is seldom mentioned in more update work in a similar vein that does not presume specific constraints on code-switching (e.g., González-Vilbazo and López, 2011, 2012; Shim, 2013, or the papers in MacSwan, 2014). No matter what specific theory or version of a theory it is, portmanteau sentences are problematic for the Null Theory, because they are radically different from monolingual phenomena, a construction that presumably arises out of language contact and hence is specific to code-switching (Chan, 2009, also refer back to the above section on differences between the portmanteau construction and monolingual doubling). This is not to suggest that theories of monolingual syntax can never be extended to the syntax of portmanteau constructions, but the “bilingual element” that sets apart monolingual sentences and the portmanteau ones has to be identified and captured in any satisfactory account of the latter.

Though put forward by a veteran in generative linguistics, the Bilingual Speech Model (Muysken, 2000, 2013) presents a rather different vision from that of the more recentstudies which continue to explore possible constraints on a specific dataset or language-pair with reference to facets of the Minimalist framework (e.g., González-Vilbazo and López, 2011, 2012; Shim, 2013). More comprehensive and “variationist” in outlook, the Bilingual Speech Model (Muysken, 2000, 2013) envisages different strategies with which bilinguals or bilingual communities engage in “code-mixing” or intra-sentential code-switching. *Alternation* refers to a total switch to another language in lexis and grammar, whereas by *insertion* a word or a phrase is inserted to a sentence framed by the Matrix Language. *Congruent lexicalization* is a third strategy where a code-switched sentence has a structure shared between the two participating languages and so words may be drawn from either language anywhere in the sentence without constraint. The fourth one, namely, *backflagging*, is the latest addition (Muysken, 2013), in which a bilingual speaker uses some elements of his/her heritage language even though he or she has shifted to a new language. In this framework, Muysken (2000, p. 104–105) does describe the portmanteau constructions, which he calls “doubling,” as *alternation*. This proposal is seconded by Takagi (2007) who renames the portmanteau construction as “symmetrical sentences” with reference to her dataset of Japanese-English code-switching produced by bilingual children. Namba (2012a,b) follows suit, but elaborates that portmanteau constructions are better treated as alternation and triggering (Clyne, 1987). For instance, in English-Japanese code-switching, a switch from an English verb to a Japanese object triggers Japanese grammar and eventually the doubling of a verb in Japanese [e.g., (2)]. However, alternation, which implies a long element after a switch, is problematic in capturing cases where there is only one word after a switch [e.g., (6), (11), (18), (19)], since the single switched word does not clearly show that the sentence switches to another “grammar.” Even though the speaker code-switches to a longer fragment, alternation may still be awkward in describing examples where there are further switches after a speaker has alternated once [e.g., (12), (16), (17), (20)], since alternation denotes a “total” or “complete” switch in lexis and grammar [as illustrated in (2), (3), (7)–(10)]. Defined as extensive code-switching in a structure shared by both languages, congruent lexicalization (Muysken, 2000) does not apply to the portmanteau construction which involves contrasting word orders from both languages. Defined as occasional switching to a heritage language that the bilingual speakers seldom use in daily life, backflagging (Muysken, 2013) does not seem to apply to the portmanteau construction either, since the bilingual speakers who produce them do seem to be using both languages actively (if not equally actively) in their life.

The optionality of portmanteau constructions (i.e., non-portmanteau constructions, e.g., SVO or SOV, are also found in code-switching with typologically different languages) appears to invite an account along the lines of Optimality Theory. In the literature, however, there are not many studies of code-switching employing an Optimality-Theoretic framework. Among these few studies, Bhatt (1997, 2014) proposes that there are different constraint-rankings for code-switching involving different language-pairs. It is hence unclear how he may account for variant patterns of code-switching involving the same language-pair, such as portmanteau vs. non-portmanteau constructions. Focusing on Cantonese-English code-switching in a PP, Leung (2001) suggests a constraint-ranking which governs possible output of constructions. In brief, he concludes that the portmanteau construction (i.e., a PP involving an English preposition and a Cantonese postposition) and the non-portmanteau one (i.e., PP containing only the English preposition) are both allowed but other possible structures are forbidden. While the account successfully captures the empirical facts, the idiosyncrasy of the portmanteau pattern and its emergence remain opaque.

## Portmanteau constructions, phrase structure and linearization

To account for the portmanteau construction, a fundamental issue that needs to be addressed is what kind of structure it may have. Apparently, the phonetic realization of two heads sharing the same complement suggests that the phrase may be ternary-branching rather than binary branching, which has been a mainstream assumption in generative grammar, particularly with reference to the Antisymmetry thesis proposed by Kayne (1994, 2004, 2005, 2009)^18^.

(45) 
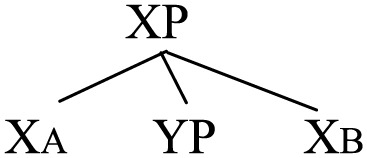


One could argue that the portmanteau phrase XP may be derived as follows in accordance with Antisymmetry and binary branching; that is, YP follows X_B_ and then it moves up before [YP X_B_] merges with X_A_.

(46) 
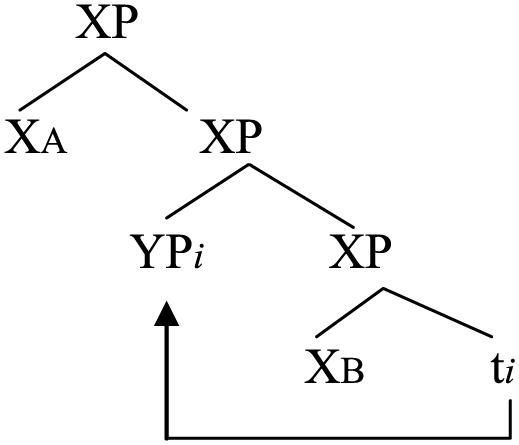


The derivation in (46) is of course very much simplified. Firstly, in all data of portmanteau constructions involving the verb (e.g., (2), (3), (6)–(14) above), the doubled verb is the *main verb* of the sentence inflected for tense and agreement. Accordingly, the derivation involves the doubling of not only V but also T, as sketched below:

(47) 
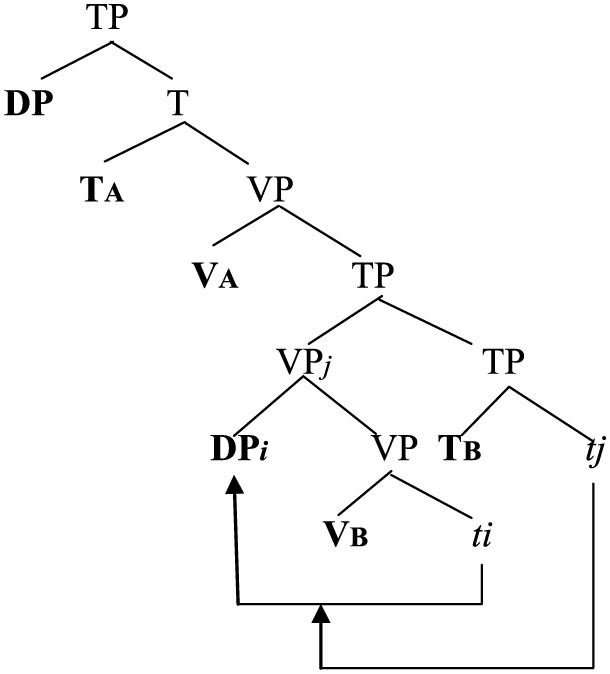


(A = a head-initial language; B = a head-final language)

In case the doubled head is a ditransitive verb [e.g., (6)] or a “saying” verb which takes a DP and a clausal object [e.g., (9)], more layers of *v*P shells (Chomsky, 1995) have to be invoked between T and V, resulting in even more derivational steps than in (47)^19^.

Several questions then arise. Are these derivations absolutely necessary? Is there a more economical way of capturing the portmanteau construction? Additionally, within the minimalist architecture of grammar, an outstanding question is why two words of identical meaning are simultaneously introduced to the Numeration (Chomsky, 1995). No matter what the answers to these questions are, there is a sense that they may well lie outside “syntax proper,” even though the portmanteau constructions, as illustrated and argued in this paper, show recurrent syntactic patterns that are subject to structural constraints.

Difficulties to account for the portmanteau constructions in generative grammar suggest that these structures may be better handled by alternative models of grammar whose assumptions are radically different, for instance, functionalist theories such as Cognitive Grammar (Taylor, 2002; Langacker, 2008) or Radical Construction Grammar (Croft, 2001). However, this does not appear to be the case. Briefly speaking, these grammars focus on the meaning of constructions which are not seen as being built up by derivations, and the language faculty is not autonomous but connected to other cognitive functions or faculties. In these frameworks, the portmanteau sentences would convey some meanings that are distinct from those of their non-portmanteau counterparts. Nonetheless, this is far from clear in the data and their descriptions in the relevant literature. A related issue is that, if portmanteau constructions do not convey some additional or different meaning, these sentences would violate the principle of economy (Haiman, 1983, 1985; Croft, 2002; Chan, 2009).

## Toward a mixed account of syntax and processing

In an innovative account, Hicks (2010, 2012) suggests that a bilingual accesses two sets of syntactic information and projects a dual structure for the portmanteau constructions, borrowing Sadock's (1983, 1991) Autolexical Syntax. A portmanteau phrase would have a structure as (48) with an upper layer and a lower layer.

(48) 
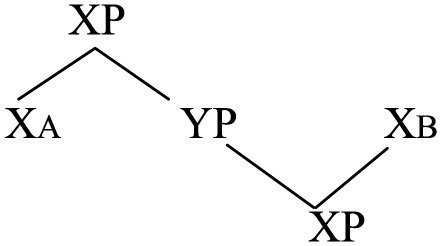


More elaborately, an SVOV sequence would have the following structure under this account.

(49) 
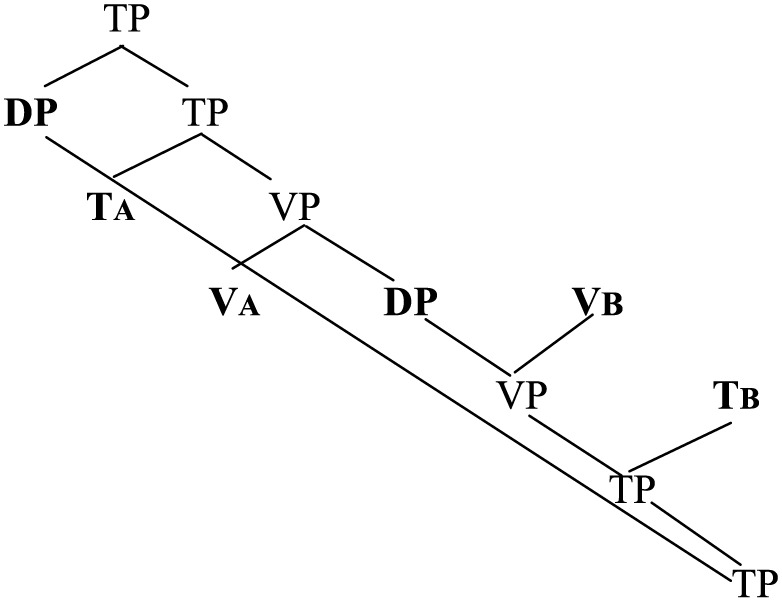


(Adapted from Diagram 3 in Hicks, 2012, p. 52).

The idea that bilinguals have access to two sets of syntactic information is intuitively convincing and uncontroversial. There is much psycholinguistic evidence that when a bilingual speaker processes or produces one language (i.e., the “target” language), the other language is also accessed (i.e., the “non-target” language, see Wu and Thierry, 2010 for an overview). However, co-access of syntactic information itself is too general a factor to explain the constraints on portmanteau constructions and the optionality of them. The constraints suggest that only certain types of syntactic information are responsible for the production of portmanteau constructions, and the optionality implies that there is a mechanism to filter out one language or one set of syntactic information, hence leading to non-portmanteau constructions in output.

The first issue is quite straightforward. It is empirically clear that portmanteau constructions emerge in language-pairs in which head-complement order is different for a particular phrase (e.g., VP, TP, CP, PP, DP). Additionally, it looks very plausible that only *projecting heads* take part in portmanteau constructions. Heads which arguably do not project (i.e., (first-)merging its complement), such as nouns, adjectives or conjunctions, do not take part in portmanteau constructions. Crucially, doubling of heads is highly related to *projection* (i.e., X merges with YP and results in an XP, i.e., [_XP_ X YP]—Chomsky, 2013). Complements (e.g., YP) do not project (i.e., ^*^[_**YP**_ X YP]), and thus they do not double (i.e., ^*^[_YP_ YP X YP]).

The second issue calls for a distinction between *access* to syntactic information and *activation* of syntactic information (i.e., the syntactic information is processed, leading to an output, a phrase or a sentence). Presumably, bilinguals always have access to information of both languages, but they do not always activate both sets of information, for instance, when they are using only one language. This is consistent with the model of Language Modes (Grosjean, 2008, etc.) in which bilinguals may activate just one language with the other deactivated (i.e., the Monolingual Mode), or they may activate both (i.e., the Bilingual Mode). Level of activation nonetheless is relative and hence the Monolingual Mode and the Bilingual Mode are two ends of a continuum, and the mode of a bilingual is affected by many performance factors such as the context of speaking, the other participants, his or her language proficiency, etc. An alternative conception is suggested in Green and Li's (2014) model, in which two languages are always active in the mind of bilinguals who engage in code-switching, but only some information is selected for output and other information is inhibited through some “control” mechanism (Green, 1986). In what Green and Li (2014) call “Competitive Control,” a bilingual may speak in one language only, and information of the other language (e.g., words and morphosyntactic rules) is inhibited. In other contexts, a bilingual may engage in extensive code-switching, exercising less inhibition and allowing information of both languages to be processed further for output; Green and Li (2014) describe this cognitive process as “Open Control.”

In Green and Li (2014) model, types of information about a language include word forms and syntactic constructions (or, more technically “Combinatorial Nodes”), and they are all linked in a network. Assuming that head-initial and head-final orders are two of such “combinatorial nodes” (i.e., [X YP] and [YP X] respectively), bilinguals of typologically different languages always have access to both sets of head-complement orders. However, portmanteau constructions (e.g., SVOV) arise when bilinguals do not inhibit either set in output. When they let only one set of order enter output (with the other inhibited), non-portmanteau constructions (e.g., SVO or SOV) would be the result.

This way of capturing linearization in the production process may seem uneconomical and a radical departure from more mainstream accounts which envisage a more direct mapping between syntactic structure (i.e., a syntactic tree) and word order (e.g., Kayne, 1994, 2004, 2005). However, it is not inconsistent with the more recent view that linearization is a process at the Sensory-Motor (SM) interface (alternatively known as the interface of Phonetic Form/PF), and that syntactic structures are not specified for linear order in derivations (Chomsky, 1995, 2005, 2013; Kremers, 2009, 2012). With reference to the portmanteau construction, locating linearization in the production process avoids complexity of structure and derivations which plagues an Antisymmetry approach and to some extent Hicks' (2010, 2012) dual-structure account^20^.

Let us further assume that a lexical item that enters a Lexical Array or Numeration and then syntactic computations is actually a bundle of related information about a word, including meaning, syntactic information (e.g., word class) and morpho-phonological information, largely equivalent to what is called a *lexical entry* in the psycholinguistic literature (e.g., Levelt, 1989)^21^. It seems that Chomsky (1995, 2005, 2013) has not rigorously defined what he meant by a lexical item except the comment that it must provide a label so that the Lexical Array will recognize it as head in projection (Chomsky, 2013, p. 43), with “label” presumably referring to the word's syntactic category. At any rate, “a lexical item” cannot just refer to a “word” in a conventional sense as a pairing of a phonetic or written form and a meaning, since it is supposed to enter syntactic computations when it has not yet been transferred (for pronunciation or writing) in the minimalist architecture of grammar. In sum, it is not inconceivable that a lexical item is a bundle of connected information about a word. Furthermore, in bilinguals' lexicon, a lexical entry consists of two phonetic forms^22^. With reference to example (2) above, this conception of “a lexical item” or a lexical entry for a bilingual lexicon is sketched below in (50):

(50) 
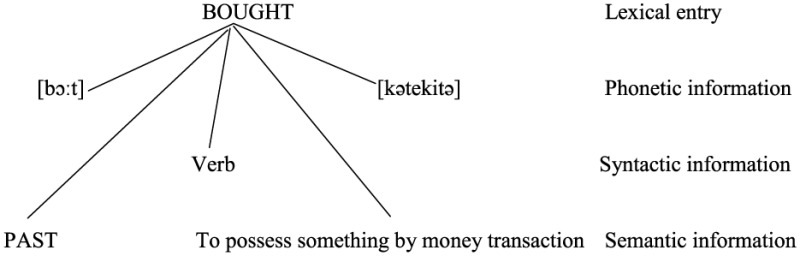


When the bilingual speaks English, the head-initial word order is selected, and so is the phonetic form [bɔ:t]. The speaker also has access to the corresponding form [kətekitə], but this information is inhibited [i.e., (51a)]. Conversely, when speaking Japanese, the head-final order is selected, calling for the form [kətekitə]. At the same time the speaker has to inhibit the corresponding form [bɔ:t] [i.e., (51b)].

(51) 
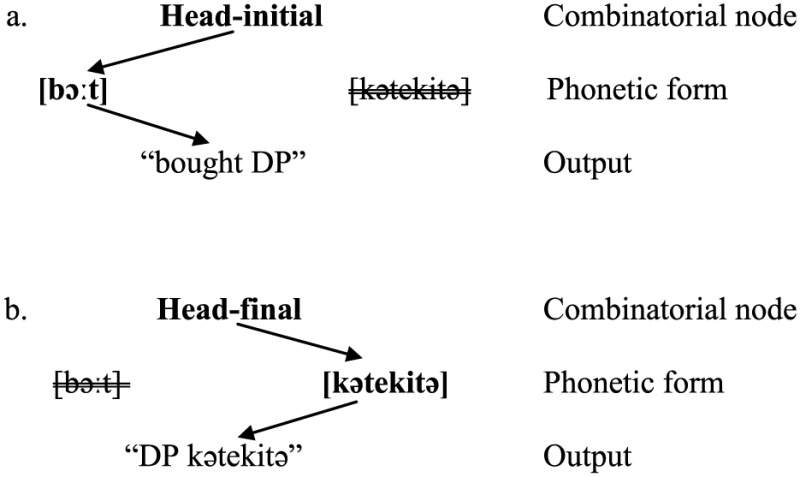


It is reasonable to assume further that [bɔ:t] is more often or strongly associated with the head-initial order whereas [kətekitə] is more strongly associated with the head-final order, and the two phonetic forms are linked between themselves by virtue of the fact that they are synonymous and belong to the same lexical entry [i.e., (52)].

(52) 
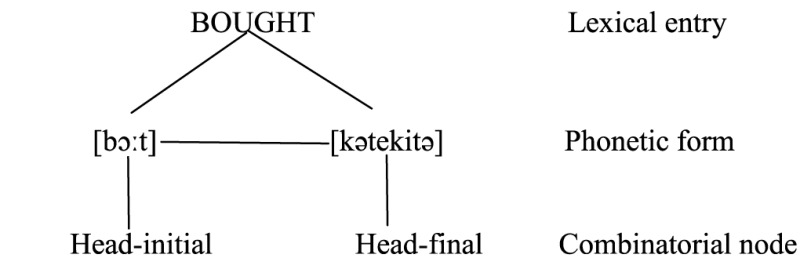


In a code-switching context, a bilingual may let both phonetic forms and both sets of word orders enter output without inhibiting either. Here comes a very important issue. Are portmanteau constructions triggered by activation of both word orders (or “combinatorial nodes”) or that of both phonetic forms? The former is much more plausible, if we assume that the bilingual mind is organized in the same way irrespective of the languages a bilingual speaks. That is, in case bilinguals speak both head-initial or both head-final languages, the activation of both phonetic forms would lead to sequences of SVVO or SOVV (or XXYP/YPXX when the doubled head is not a verb). Judging from the absence of these sequences (until they are documented in future), it appears that bilinguals do not usually activate both phonetic forms, even though this is actually possible under the Bilingual Mode (Grosjean, 2008). Therefore, portmanteau constructions are more likely to be motivated by the activation of both word orders which in turn call for the two corresponding phonetic forms.

(53) 
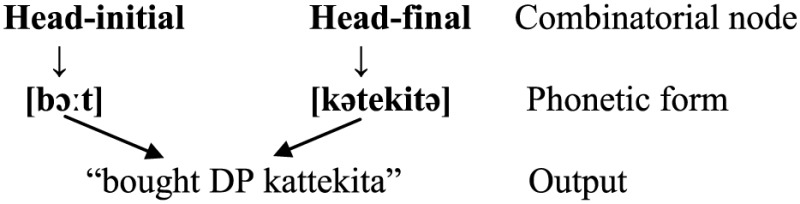


All the processes described in (51) and (53) are supposed to take place at the Sensori-Motor (SM) interface, the place where words are put in linear order and instructions are sent to the vocal organs to pronounce the words (it was called Phonetic Form (PF) in Chomsky's (1981, 1995) earlier works, largely equivalent to the stage of “planning” in language production models (Green and Li, 2014); in other words, the syntactic structure underlying the sequences of VO, OV (i.e., the non-portmanteau constructions) and VOV (i.e., the portmanteau constructions) is actually the same VP with the relative order of V and object DP unspecified.

(54) 
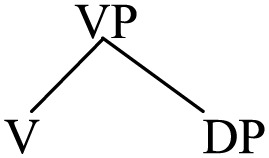


Generalizing this to portmanteau constructions where the doubled heads may not be a verb, the underlying structure of them is not exactly [X_A_ YP X_B_] as represented in (1) but simply an XP, with order between X and YP unspecified. Duplication of X_A_ and X_B_ arises in the Sensori-Motor interface as a bilingual activates both sets of head-complement order and realizes them with synonymous forms in two languages. Despite different phonetics these two forms are actually the *same* word belonging to the *same* lexical entry.

(55) 
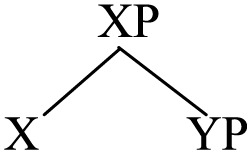


## Remaining issues

There are a number of residual issues to be tackled. Firstly, the proposal so far has not fully explained the empirical fact that the language of the head always determines head-complement order in portmanteau constructions, especially when we consider that in non-portmanteau code-switched sentences the language of the verbs does not always determine head-complement order [see (39)–(44) above]. On the other hand, the language of functional heads (including adpositions) does seem to determine head-complement order in portmanteau and non-portmanteau sentences (Chan, 2003, 2008, 2015). The problem here is not so much about the portmanteau construction itself which is in a way explained by the proposal. The issue is really about how to explain verbs whose linear order does not match its “language” [i.e., a verb from a VO language appears in OV order or a verb from an OV language appears in VO order, e.g., (39)–(44)]. In addition, how do we account for the asymmetry between the verb and the other categories (i.e., C, I/T, D, also P tentatively) the language of which always determines head-complement order in portmanteau or non-portmanteau contexts?

Concerning the former issue, a recent syntactic account suggests that the properties of VP, including VO/OV order (in code-switching or in “pure” languages alike), are dependent on the feature composition of *v* (González-Vilbazo and López, 2011, 2012) and probably another functional head Asp(ect) between *v*P and VP (Shim, 2013). Putting aside how these models can be modified to accommodate the portmanteau construction (i.e., they do not specifically aim to explain the portmanteau construction), here we attempt to extend the psycholinguistic/production approach outlined above. Recall from (51) above that when the head-initial order is selected, the default case is that a verb associated with a VO language is also selected. This phonetic form, however, can be inhibited. As the processor is fast looking for a “substitute” for that form to produce the syntactic construction, the corresponding word form associated with an OV language is selected for output [i.e., (56)]^23^.

(56) 
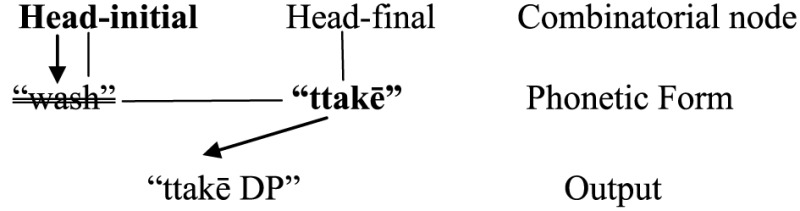


[Referring back to the English-Korean example in (40)].

Reversely, when a head-final order is selected, the default case is to activate a verb from an OV language, but this word form can be inhibited so that the corresponding word form from a VO language is selected for output [e.g., (57)].

(57) 
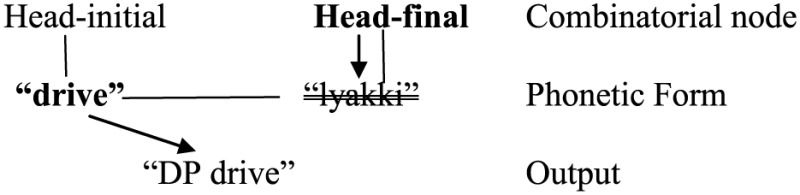


[Referring back to the Tamil-English example in (44)].

Under a psycholinguistic approach pursued here, the reason why a form is inhibited is prompted by performance factors including processing (e.g., the corresponding word form associated with another language has been more active in the context of speaking or “triggered” by a related form—Clyne, 1987, etc.) or pragmatics (e.g., that word form is deemed more appropriate in the context, i.e., the “mot juste,” see Footnote 1).

These patterns [e.g., (39)–(44)] may well arise in a mental state between Competitive Control (Green and Li, 2014), where one language is selected and the other is inhibited, and Open Control (Green and Li, 2014), where information of both languages is allowed to enter output. In other words, a bilingual is speaking a selected language and yet the non-selected language is not completely blocked, and so some elements of the non-selected language may be selected for output. This is a state which Green and Li (2014) call Co-operative/Coupled Control.

In the case of functional heads, we may conjecture that their phonetic form is strongly associated with a combinatorial node and so it cannot be inhibited. Consequently, the mismatch between the language of a head and head-complement order [e.g., (56) and (57)] is not possible.

When both head-initial and head-final orders are activated, as in the case of portmanteau constructions, a bilingual presumably exercises less inhibition of information from both languages, that is, a state which is described as Open Control (Green and Li, 2014). Accordingly, both phonetic forms are activated without suppression of any one of them and they will go into their default position; for instance, the verb form associated with a VO language always goes into its default pre-nominal position and likewise its corresponding form associated with an OV language always appears post-nominally. The condition for the processor to find a “substitute” [i.e., the default word form associated with a combinatorial node is inhibited, as in (56) and (57)] does not exist anymore.

Now we turn to syntactic issues. The current proposal suggests that projection of a phrase triggers transfer and then linearization; however, there are two alternative scenarios as to the timing or the transfer. That is, either transfer is kickstarted as soon as a projecting head (first-)merges its complement along the lines of Kremers (2009), or it proceeds in *phases* in which a sentence is spelt out successively in *v*P and CP (Chomsky, 2005, 2013; Citko, 2014; also adopted in González-Vilbazo and López, 2011, 2012 and Shim, 2013 for code-switching). It is the standard phase theory which seems to provide a more unified explanation of portmanteau constructions involving different categories of a reduplicated head. More precisely, the “immediate linearization” approach can apparently explain portmanteau CPs [C being doubled, e.g., (8)], IP/TPs [I/T being doubled, e.g., (15)] and PPs [P being doubled, e.g., (17)], DPs [D being doubled, e.g., (21)], those instances in which verbs are doubled, which appear to be more common, suggest that linearization is procrastinated before it is transferred to the SM interface for linearization. That is, as these doubled verbs [e.g., “*bought*” in (2)] are morphologically inflected, they are supposed to move up to higher functional heads, for instance, the *v* head which selects VP (González-Vilbazo and López, 2011, 2012; Chomsky, 2013). The reason why the verb, contrary to other kinds of heads, has to undergo further derivations is partly morphological and partly semantic (i.e., a verb takes up morphological marking to encode information such as tense, aspect and agreement). On the other hand, even though the other kinds of heads involved in portmanteau constructions do not seem to undergo further derivation or movement, it is not necessary that they must be transferred and linearized as soon as they project a phrase with their complement.

Thirdly, the copula verb may be doubled in the portmanteau construction, but it is not unanimously agreed that the copula projects a VP or copula phrase with its complement. In recent works, a copula verb merges with a small clause [XP YP] and XP raises eventually (e.g., [XP COP [XP YP]]; Moro, 2010; Chomsky, 2013). There is however an alternative account in which the copula verb does project a phrase as a Relator head and merges with a predicate phrase as its complement (den Dikken, 2006). This account appears to be more consistent with this account of the portmanteau construction.

## Conclusions

This paper proposes a combined syntactic and psycholinguistic account of portmanteau constructions in code-switching. The syntax side of the account crucially hinges upon the minimalist view that order is an interface phenomenon but syntactic structures (Chomsky, 1995, 2005, 2013; Kremers, 2009, 2012), at least those of a phrase in which a head merges a complement, are not specified for order. One other assumption needed is that a lexical item which enters into a Lexical Array and eventually syntactic derivations is actually a “lexical entry” which is a bundle of various kinds of information about a word. In the case of bilinguals, this lexical item also contains information of a word in two languages. The psycholinguistic side of the account relies on Green and Li's (2014) model of Cognitive Processes of Control in which bilinguals may select one language for output and inhibit another, or they may let information of both languages be processed further for output. Crucially, projection of a phrase will lead to linearization, and a bilingual may co-activate and process both word orders (i.e., head-initial and head-final) if he or she speaks a head-initial and a head-final language. Whereas there is much work to be done to further clarify a number of issues pertaining to the account (in particular, whether the activation of both word orders is intentional or due to lapse of inhibitory control), this paper discusses an interesting case in which a limited set of performance data of a language-contact phenomenon, that is, the portmanteau construction, could lend empirical support to the ideas that a syntactic object is order-less and linearization is a process at the Sensori-Motor interface. At any rate, it is hoped that this work, despite all its limitations and stipulations, will raise more scholarly interest in the portmanteau construction and related issues, and stimulate more research on these topics.

### Conflict of interest statement

The author declares that the research was conducted in the absence of any commercial or financial relationships that could be construed as a potential conflict of interest.

## Footnotes

^1^There is an issue of whether there are cross-linguistic synonyms or “translation equivalents” which are really “synonymous” with exactly the same meaning in terms of reference, style or connotations. In the code-switching literature, it has indeed been suggested that code-switching of a single word or a short phrase is motivated by the fact that this word or phrase conveys nuanced meanings which are not conveyed by its synonym or translation (e.g., Li, 2001; Curcó, 2005). In other words, the code-switched word or phrase is incurred by virtue of being the (more) appropriate expression or the “mot juste” (Gafaranga, 2000). Nonetheless, if we consider that the meaning of a word is fuzzy and fluid, being adjusted and fine-tuned in different contexts (Wilson and Carston, 2007), it is plausible that the cross-linguistic synonyms are intended or treated as “exact equivalents” in some contexts. For instance, on fairly formal occasions where only one language is expected, a bilingual speaker may code-switch to a word which is the mot juste (Gafaranga, 2000) but immediately afterwards provides a synonymous translation of that word in the language that is supposed to be the language in the ongoing interaction. In these acts of “medium repair” (Gafaranga, 2000), the code-switched word and its translation are arguably intended and treated as equivalent and identical in meaning, even though in other contexts the bilingual speaker may use the two words to express nuanced meanings. In the context of portmanteau constructions, it is plausible that the doubled words are treated as equivalents with identical meaning. That is, the use of two (near-) synonymous forms from both languages is not so much due to the expression of nuanced meanings, but it is motivated syntactically to satisfy both the head-initial and head-final orders which are co-activated (see below for more details). The synonymy or equivalence of the doubled words tallies with the intuition of many authors of the papers who are also bilinguals interacting with those producing the code-switched, portmanteau constructions, as shown in their glosses and English translations of the examples (e.g., Nishimura, 1985, 1986, 1995; Park, 1990; Namba, 2012a,b). In some rare cases where the doubled words are quite different in meaning and hence not synonymous, these authors were able to notice and indicated that accordingly in the glosses and translations - see (4) and (5). Besides, there is some psycholinguistic evidence that forms of cross-linguistic synonyms or translations are co-activated when a certain meaning (or lemma) is activated (e.g., *dog* and *perro* for a Spanish-English bilingual) to the point that a cross-linguistic synonym (e.g., *perro*) facilitates the access and production of *dog* in picture-word-interference experiments (Costa et al., 2000; Runnqvist et al., 2013).

^2^See example (6) below in which a ditransitive verb is doubled and the shared element consists of two objects or phrases.

^3^In fact, neither Hicks (2010) nor Stolz (1996) makes this explicit. Stolz (1996), however, does imply this as he glossed “−*sti*” with an abbreviation “COO,” and elsewhere only the bound morphemes seem to be glossed with an abbreviation. With a view that conjunctions are rarely clause-internal, which appears to be the case for “−*sti* ” in (23), I assume that “−*sti*” is a bound morpheme. At any rate, the point here is that it is possible to find doubling of synonymous elements involving a word and a bound morpheme.

^4^For instance, there are more or less the same number of VO or OV languages in the world (Dryer, 2013b), but cross-linguistically suffixes seem overwhelmingly more prominent than prefixes, and infixes are much less common (Dryer, 2013a).

^5^As for the portmanteau constructions, the authors, many of whom are bilingual in the two languages involved, appear to interpret the doubled elements as synonymous and equal in meaning, which is shown in the glosses and English translations (e.g., Nishimura, 1985, 1986; Park, 1990); see footnote 1 above.

^6^See below for more discussion about a movement analysis of portmanteau sentences.

^7^“Portmanteau” from Wikipedia (http://en.wikipedia.org/wiki/Portmanteau).

^8^“Palindrome” from Wikipedia (http://en.wikipedia.org/wiki/Palindrome).

^9^That is, there may be more deep-rooted similarities between monolingual syntactic doubling and portmanteau constructions which this section has not addressed and which are open to further research.

^10^Grammaticality judgment of code-switched sentences has been widely assumed to be affected by bilinguals' varying proficiency in their two languages. Toribio (2001) finds that a group of Spanish-English bilinguals of different proficiency levels show varying grammaticality judgments. Other researchers were prudent in choosing only competent bilinguals to be their subjects (e.g., MacSwan, 1999; González-Vilbazo and López, 2011, 2012). The point here is not to suggest that grammaticality judgment is never valid in code-switching studies, but it may be problematic to ask different groups of bilinguals (who speak various language-pairs) to give grammatical judgments of portmanteau sentences, including patterns attested in the data and other hypothetical ones.

^11^Attributive adjectives are standardly assumed to be an adjunct but not a head (Santorini and Mahootian, 1995), but some scholars do take attributive adjectives to be a head (Cantone and MacSwan, 2009).

^12^Haspelmath (2007) points out that [XP YP CONJ] does exist in some languages, for example, *que* in Latin (Haspelmath, 2007, p. 8), but it is much rarer than an [XP CONJ YP] sequence cross-linguistically. Even in the exceptional case of the conjunctive morpheme in (23), it is more likely to be clause-initial in syntactic derivation but eventually gets affixed to the subject noun via lowering at PF (Embick and Noyer, 2001). The possibility that it is merged in clause-final position seems far more remote.

^13^Apparently, an SOVO structure would also violate the head-initial/head-final value parameterized for the language of the verb. However, the picture is more intricate in code-switching in which the language of the verb does not necessarily determine head-complement order (Chan, 2003, 2008, also see below).

^14^Even if the doubled verbs are seen as two verbs, it is not problematic to assume that the subject and object of portmanteau sentences receive two theta roles. In monolingual syntax, arguments in complex predicates or the subject in control sentences seem to receive two roles too, but from two different verbs or predicates (Ackema, 2014).

^15^See chapter 7 in Muysken (2000) more in-depth discussion of the mixed compound verbs.

^16^Note that portmanteau constructions do not necessarily violate the Equivalence Constraint if the switch takes place within YP (e.g., X_A_ [YP] X_B_) the internal order of which is shared between language A and language B (e.g., [_DP_ D ADJ N]). However, in most existing data [i.e., (2), (3), (6)–(18)], the switch falls either between X_A_ and YP or between YP and X_B_, thus contravening the constraint.

^17^Apparently not having touched upon the portmanteau constructions so far, Myers-Scotton (1993, 2002) did tackle examples of morphological doubling [e.g., doubling of the plural affix in (22)]. The explanation is that, as a bound morpheme is retrieved from ML [e.g., the Shona plural affix “*ma*” in (22)], its counterpart in EL [e.g., the English affix “−*s*” in (22)] is also co-activated, resulting in morphological doubling. Even though this apparently violates the System Morpheme Principle (i.e., a bound morpheme, supposedly a “system morpheme,” is drawn from EL), the grammar of ML is still respected with the doubled morpheme from ML. The idea was formalized as *the Double Morphology Principle* in Myers-Scotton (1993, p. 133). Under the “4M” Model, the appearance of an EL system morpheme is even less of a problem (Myers-Scotton, 2002, p. 91–93) so long as it is not an “outsider” system morpheme. Putting aside the desirability of such reasoning (i.e., more machinery is invoked to deal with apparent counter-examples, see Chan, 2003, 2009) it seems difficult to apply similar argumentation to the portmanteau construction (i.e., a head from EL is allowed as long as the corresponding head from ML is lexicalized). Crucially, the doubling of heads involves word order, hinging upon the Morpheme Order Principle. Additionally, many examples do involve a switch to longer elements or more than one instance of syntactic doubling [e.g., (2), (7)–(9)] so that it does look virtually impossible to assign an ML and an EL for that code-switched sentence.

^18^Ternary or “flat” structures are allowed in Simpler Syntax (Culicover and Jackendoff, 2005), however.

^19^In current phase-based theory (Chomsky, 2013; Citko, 2014), the light *v* is always introduced into the derivation of a sentence.

^20^There may be two more limitations of Hicks' (2010, 2012) dual-structure account. Firstly, it is not apparent that the doubled heads are of one word (see text above), since they are dominated by different layers of structure. This also leads to the second drawback; that is, the dual-structure does not show that a [X YP X] sequence is intuitively one phrase. However, the dual-structure account may well capture cases in which the doubled heads are two different words with different meanings [i.e., (4) and (5)].

^21^Whereas “lemma” seems a more familiar term used to refer to information about a word, according to Levelt (1989), it does not include the morpho-phonological form of a word, and hence I use “lexical entry” instead in this paper.

^22^There is some psycholinguistic evidence that the two cross-linguistic synonyms or translations are both highly co-activated when a concept or *lemma* is activated (Costa et al., 2000; Runnqvist et al., 2013). See footnote 1 above.

^23^This account does not address the issue of why a verb from a VO language is often accompanied by a light verb see (43) and (44) when it appears in OV order. See alternative accounts in González-Vilbazo and López (2011, 2012) and Shim (2013).
